# Long term porosity of solid electrolyte interphase on model silicon anodes with liquid battery electrolytes

**DOI:** 10.1038/s42004-024-01381-2

**Published:** 2024-12-19

**Authors:** Jonas Grill, Jelena Popovic-Neuber

**Affiliations:** Department of Energy and Petroleum Engineering, University of Stavanger, Stavanger, Norway

**Keywords:** Batteries, Batteries

## Abstract

A stable solid electrolyte interphase (SEI) is of great importance for battery electrodes in terms of cycling as well as for its shelf life. While SEI formation on silicon anodes is generally only studied after the first charge and discharge of cells and initial reaction of electrolyte, we show the formation of a liquid/solid SEI in symmetric cells with silicon electrodes in contact with carbonate and glyme-based electrolytes under close to open circuit conditions and its behavior during long-term ageing. Activation energies of SEIs were measured via temperature-dependent electrochemical impedance spectroscopy (EIS) to study the contribution of liquid/solid phases to ion transport. The effect of different solvents, salts, their concentrations, and final water content of the glyme-electrolyte on the SEI was studied in detail. SEIs formed in cells with glyme-based electrolytes are generally more porous than the ones in cells with carbonate-based electrolytes. The addition of vinylene carbonate to glyme electrolyte is shown to be beneficial for its SEI, as it causes lower and more stable SEI resistances over time. A small amount of water in glyme electrolytes causes a denser SEI without much change in SEI resistance.

## Abstract (web summary)

A stable solid electrolyte interphase (SEI) is of great importance for battery electrodes for charging/discharging purposes, but the mechanism of SEI formation is not fully understood. Here, the authors study the formation and long-term evolution of the SEI near open circuit conditions in symmetric silicon cells containing different electrolyte chemistries.

## Introduction

High power and energy density is a crucial metric for next-generation batteries, as current commercial lithium-ion batteries are limited by the low specific capacity of their graphite anodes (370 mAh g^−^^1^)^1^. Silicon anodes have been pinpointed as promising materials for future battery technologies since 1970s due to their high theoretical specific capacity of 4200 mAh g^−^^1^, about ten times higher than graphite^2–4^. In addition, in the last years, silicon has been broadly used in the form of carbon/Si composite material, where this high theoretical capacity has been linked with the high stability of the graphite electrode^5^.

The SEI is an organic/inorganic passivation layer between the corresponding anode and electrolyte formed by (electro)chemical decomposition of the electrolyte upon direct contact with the electrode^6^. It is a major factor for the electrochemical performance of batteries (power capability, shelf- and cycle life) and has to offer an effective cation transference number close to unity while being also electrically insulating, to prevent further decomposition of the electrolyte^7^. In principle, well-performing SEI is only several nm thin, kinetically and mechanically stable, and causes negligible resistances in full cells due to its suitable ionic conductivity^8,9^.

General understanding of SEI on Si electrode is that it forms during the first charging process in the lithium battery cell, and is highly dependent on the electrolyte chemistry^2,8^. The formation mechanism proceeds via decomposition of the electrochemical double layer and a series of possibly coupled (electro)chemical reactions. As the kinetics of these reactions encompass multiple time and length-scales, a holistic view of SEI formation is often not feasible. For a detailed review on the impacts of solvents, salts and additives on the SEI, the reader is referred to ref. ^10^. A suitable SEI specifically designed for silicon electrodes has to solve several challenges, the most important one being volume expansion of up to 400%, causing considerable mechanical stress^2^. This may promote the growth of a detrimental SEI if it cracks, exposing fresh Si to the electrolyte and leading to its further consumption, causing active lithium inventory loss. A thinning of the SEI can also be problematic, as it may allow electrons to pass through^6^. On the other hand, an increase of the SEI thickness leads to the increase of Li^+^ diffusion distances and total resistance, as well as decrease in rate and cycling performances. This may further exacerbate the problem of slow Li^+^ diffusion in Si^2,11^. It has been shown that thicknesses of SEI as high as up to 2.5 μm are possible after 300 cycles in commercial pouch cells with unknown electrolyte, that the SEI structure is complex and multi-layered, and that SEI formation is highly temperature dependent^12^. This of course means that under such circumstances, Li inventory loss is too high for commercial applications. SEIs on Si have also been observed to be incontinuous, with island-like morphology consisting of insoluble lithium salts on the electrode surface^13^. In addition, SEI components may dissolve into electrolyte at elevated temperatures^14^. Such dissolution can beselective as the one shown for unwanted carbonate- and oligomer SEI components via addition of gamma-butyrolactone^15^. This, however, may negatively influence the ionic conductivity, as insoluble salts such as LiF and Li_2_O are poor ionic conductors^16,17^.

Thin native oxide layers are expected to exist on silicon electrodes. The growth and thickness of such native oxide films on Si wafers are known and well researched due to their importance for semiconductor manufacturing. Depending on the surface treatment and air humidity they are dense films, with a thickness that ranges from roughly 2 to20 Å^18,19^. However, chemical stability of such native passivation layers with battery electrolytes is so far widely unknown.

As the SEI is a highly complex, thin, and buried interface, its studies are experimentally challenging and require the combination of a variety of methods, as well as preferably *operando* or in situ insight to gain a full picture. The chemical composition of the SEI in carbonate electrolytes has been determined by X-ray photoelectron spectroscopy and reported to contain lithium silicate (Li_x_SiO_y_), lithium carbonate (Li_2_CO_3_), lithium fluoride (LiF) as well as lithium alkyl carbonates and related decomposition products of the electrolyte^8,20,21^. Veith et al. measured the SEI thickness and density via in situ neutron reflectometry as a function of state of charge, and observed a moderate thickness change from 180 to 250 Å during cycling^22^. Cao et al. used in situ X-ray reflectometry on native-oxide terminated Si wafer as anode, and observed a two-layered SEI with a smooth, inorganic Li_x_SiO_y_ bottom layer formed by lithiation of the native oxide film around 0.7 V and a conformally grown inorganic top SEI layer containing mostly of LiF above 0.6 V^23^. Martín-Yerga et al. showed that scanning electrochemical cell microscopy, when coupled with shell-isolated nanoparticles for enhanced Raman spectroscopy, can be a powerful high-throughput combinatorial screening tool for SEI formation under different electrochemical conditions^24^. The paper reports a continuously evolving SEI due to its dissolution in cells with carbonate electrolytes, with the SEI composition being highly dependent on the solubility of its upper organic layer^24^. Benning et al. reported a decrease of surface roughness during initial lithiation due to SEI formation on Si thin films by in situ electrochemical atomic force microscopy, followed by a drastic increase due to anisotropic lithiation of electrode particles^25^. Cryo-electron microscopy is another powerful technique to image the nanostructure and local chemistry of highly air- and beam damage sensitive SEI components, but it usually requires washing of samples as well as exposure to vacuum, when performed ex situ^26^. Dopilka et al. used nano-Fourier-transform infrared spectroscopy on amorphous Si electrodes to determine the effects of washing on the SEI with a typically used solvent, dimethyl carbonate, and observed significant morphological and chemical changes of the SEI^27^. This serves as an indication that ex situ measurements like X-ray photoelectron spectroscopy, especially the ones involving washing of the electrodes, may not accurately represent the state of the fragile SEI, while techniques such as sputtering can also introduce damage to soft or reactive SEI components. Stetson et al. used scanning spreading resistance microscopy to observe the effects of resting and temperatur change e on SEI thickness and resistance in carbonate based electrolyte and reported an equilibrium state of formation and dissolution of the upper organic layer where resting time and higher temperature caused increased dissolution^14^. McBrayer et al. monitored the surface passivation of the SEI on silicon thin films during calendar aging *via* scanning electrochemical microscopy and increased electronic conductivity of SEI over time on fully delithiated electrodes compared to fully or partially lithiated ones, indicating that chemical degradation *via* dissolution of the SEI is responsible for calendar aging^28^.

One of the straightforward, non-destructive in situ methods to study SEI formation is electrochemical impedance spectroscopy (EIS)^29,30^. EIS allows the observation of cells under near open circuit conditions, if low AC amplitudes are applied and can give deep insight into the resistances and capacitances and, when suitable cell constructions are applied and models are derived, indirectly into morphology of the SEI. Recently, we developed a methodology to investigate the growth and ion transport through SEIs on planar metal electrodes, which involves activation energy study of ion transport through SEI and development of suitable equivalent circuit models for symmetric cells^31,32^. In this work, we extend this methodology and study the behavior of symmetric Si cells with commercial Si wafers and liquid lithium battery electrolytes. Si wafers were used as a proof-of-concept model system, due to their well-defined surface morphology and chemistry. The impedance response of planar electrodes can be easily modelled, while porous electrodes composed of Si nanoparticles, may display a more complex impedance response (for a detailed discussion, see SI). Similar materials have been studied previously in Li metal half-cells using a combination of secondary ion mass spectrometry, scanning transmission electron microscopy, atomic force microscopy, and scanning spreading resistance microscopy, but not with EIS^33^. Here it has been speculated from detailed microscopy of the SEI formed in contact with a 1.2 M lithium hexafluorophosphate, LiPF_6_, in ethylene carbonate, EC/ethylmethylcarbonate, EMC (30/70 w/w) electrolyte, that initially porous structure grows in thickness over 300 cycles, where later surface heterogeneities stem from amorphization of underlying Si. We chose LP30 (1 M LiPF_6_ in EC/dimethyl carbonate, DMC, 50/50 v/v) and carbonate-based electrolytes in general, as they are used in current generation lithium batteries^34^. Vinylene carbonate (VC) was chosen as it is a common additive for commercial graphite cells which has also been successfully employed in Si-based anodes to stabilize the growth of apparently well-performing SEI with carbonate-based electrolytes^35–37^. Glyme-based electrolytes are a second electrolyte class of choice, as they have also recently been shown to improve the performance of Si anodes by enabling the formation of a conformal and robust SEI^38^. In particular at higher salt concentration and with suitable additives, glyme based electrolytes enabled stable and reversible cyclability linked with high Coulombic efficiency^39^.

In this work, we study the formation and long-term evolution of the SEI near open circuit conditions in symmetric cells containing different electrolyte chemistries by varying lithium salts and their concentration, water content in final electrolytes, and ageing times (from short-term cell ageing to up to 9 months). The detrimental effects of water content on the formation, evolution, and properties of SEI in carbonate-based electrolytes with LiPF_6_ have been demonstrated previously^40–42^. The close to open circuit voltage (e.g., small perturbation for EIS measurement) ageing behavior is of high relevance for shelf storage, both in terms of pure Si and Si-containing electrodes.

## Results and Discussion

### Short and long-term ageing of Si in contact with LP30 electrolyte

**Fig. 1:**
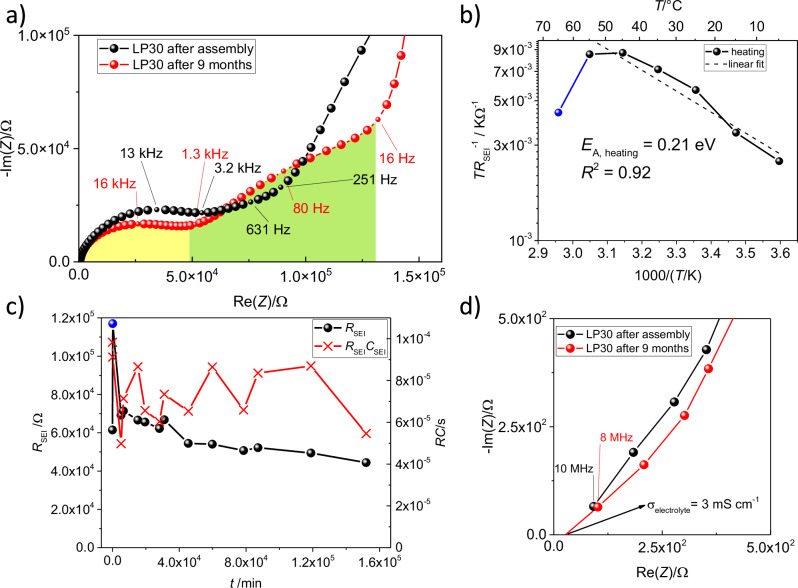
Results of electrochemical impedance spectroscopy performed on Si │ LP30 │ Si cells. **a** Nyquist plots for cells directly after assembly (black) and after 9 months of close to OCV ageing (red), yellow shade under the black curve marks the SEI semicircle after nine months, green shade marks the charge-transfer contribution. **b** Activation energy investigation of the ion transport through the interphase. Blue datapoint at 65 °C was excluded from the linear fit as it probably stemmed from accelerated cell ageing due to high temperatures. **c** Time-dependent change of SEI resistance, *R*_SEI_ (black dots), and the time constant, *RC* (red crosses). **d** Nyquist plot zoom in of (**a**) close to *x*-intercept for the first semicircle, showing the 45° slopee. The calculated ionic conductivity of the electrolyte after nine months is 3 mS cm^−^^1^.

Nyquist plot of the EIS data collected from Si │ LP30 │ Si cells directly after (ca. 5 minutes) cell assembly reveals an existence of a semicircle with *f*_max_ = 6 kHz, followed by a slop or a very depleted semicircle with a *f*_max_ = 20 Hz (black line in Fig. 1a). For comparison purposes, the capacitance of the bulk electrolyte can be calculated from the plate capacitor equation, $C=\frac{{\varepsilon }_{0}{\varepsilon }_{r}A}{d}$, as the geometry, meaning electrode distance *d*, area *A* and *ε*_r_ of the electrolyte, is known. For LP30, a bulk electrolyte capacitance of 6.3 × 10^−^^11^ F was calculated (for triglyme a slightly lower value of 1.0 × 10^−^^11^ F), leading to the conclusion that the high-frequency process (above 1 kHz) should be assigned to the ion transport through an interphase, which we will call SEI. After nine months of near-to-OCV storage (red line in Fig. 1a), in this case meaning an occasional EIS measurement for 2 h from 10 MHz to 1 mHz with 10 mV overpotential, the same features are visible. However, a decrease of the SEI resistance, *R*_SEI_, from 62 kΩ to 42 kΩ (yellow shaded area in Fig. 1a), and an increase in charge-transfer resistance, *R*_CT_, from 61 kΩ to 80 kΩ (green shaded area in Fig. 1a), indicate that the interface has changed. The total resistance of these cells is roughly two orders of magnitude higher than the one reported for porous Si electrodes of unknown chemical origin^43^. In particular *R*_CT_ is very high in this case, indicating that electron transfer is even more sluggish than the ion transport process after 9 months of ageing. The asymmetry of the observed semicircles at high frequency (i.e. depletion) is similar for both time scales of ageing and could originate from partial electronical conductivity of the SEI (“leaking capacitor” model)^44^.

The activation energy of the process was determined from the temperature vs. diameter of the semicircle arc correlation after nine months of cell ageing (detailed description is available in SI, supplementary note 1), similarly as in ref. ^24^. It should be noted that higher temperatures influence the rate of kinetically limited processes, such as SEI dissolution, as reported in ref. ^14^. Thus, temperatures above 55 °C were omitted from the investigation. Figure 1b shows an Arrhenius-type plot of *T*/*R*_SEI_, and according to the linear fit, the activation energy of the ion transport through the SEI was calculated to be *E*_A,SEI_ = 0.21 eV. This value is comparable to the *E*_A_ of liquid electrolytes and a porous SEIs in alkali metal cells^32^, where values *E*_A,SEI_ < 0.5 eV indicate the relevance of liquid ion transport processes while an *E*_A,SEI_ around 1 eV should be expected for all solid-state SEI compounds^45^. Thus, not only the capacitance, as stated before, but also *E*_A,SEI_ indicate that this is a process related to an interphase. Using this methodology, it cannot be differentiated if only the native oxide layer on the Si wafer, the SEI on top, or only parts of the SEI are porous, since all cases would cause direct contact of the electrolyte, causing the low *E*_A,SEI_ of the ion transport through the interphase. We do not expect the native oxide layer to be porous at the beginning of the contact with electrolyte, but it is not excluded that its mechanical or chemical integrity is disturbed in an electrochemical cell under pressure (e.g. coin cell).

Figure 1c shows the evolution of *R*_SEI_ related to the first semicircle (shaded in yellow in Fig. 1a) over time, and the related time constant, *τ* = *RC*, calculated from the fits of impedance data via an RC-model (see SI, supplementary note 2 and Figure S1a in SI for a detailed description of the equivalent circuit model). *R*_SEI_ increased by roughly 100% two hours after assembly and decreased slightly over the course of nine months. The time constant fluctuated around a stable value, indicating a continuous change in *C*_SEI_ and therefore of the capacitance of the solid part of the SEI. For a detailed discussion about the circuit model we refer the reader to our work on porous SEIs on alkaline metal electrodes, where capacitances are discussed in more detail^32^.

A 45° slope at the intercept can be observed for the high frequency semicircle (Fig. 1d) with capacitances lying in the 10^−9^ F range. From our understanding, such a response is either originating from (i) porous electrode due to partial electronic conductivity of the solid part of the SEI or (ii) is an artefact of measurement at higher frequencies (for a detailed discussion see supplementary note 3, SI). Such an artefact could not be observed in our previous work, since the used frequencies are 10^6 ^Hz at highest, while those features are here observed at slightly higher frequencies. As the *x*-intercept denotes *R*_electrolyte_, it can be used to calculate the ionic conductivity of the electrolyte, *σ*_electrolyte_ = 3 mS cm^−1^ (see Fig. 1d). It is of the same order of magnitude as the already reported ionic conductivity of the electrolyte *σ*_LP30_ = 12 mS cm^−1^^46^. We tried fitting temperature-dependent charge-transfer data to obtain the *E*_A,CT_ of the process, but could not obtain meaningful fits. Since the thickness of the SEI is not known in this case, we are unable to use the parallel switching model to determine the volume percent of the liquid phase. However, from the back of the envelope calculations, with assumed thickness of 100 nm, it is calculated to be 0.01 volume % for LP30 and 0.03% for 1 M LiTf in triglyme^47^.

### Effect of electrolyte chemistry on Si interphase properties: Lithium salts, their concentration, and electrolyte water content

**Fig. 2:**
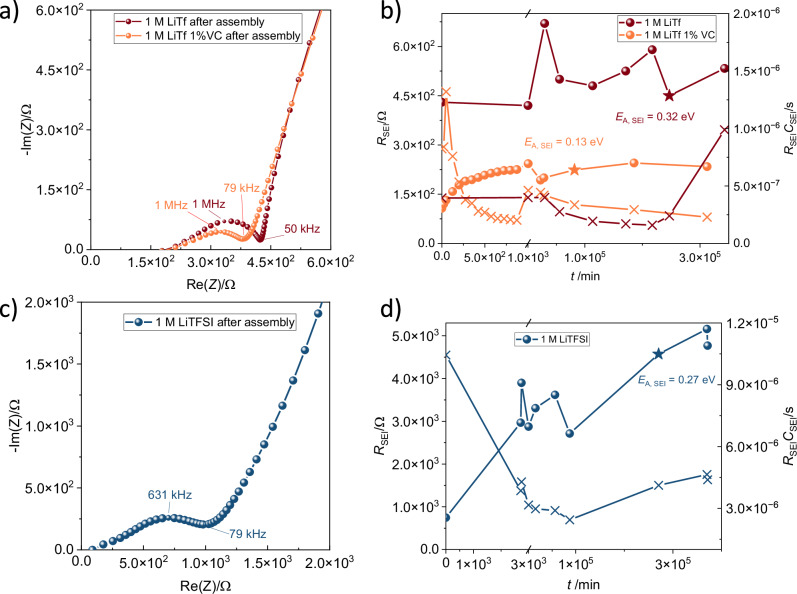
Results of electrochemical impedance spectroscopy from cells containing symmetric Si electrodes in contact with different triglyme-based electrolytes. **a** Nyquist plots for cells directly after assembly with 1 M LiTf in triglyme (brown dots) and 1 M LiTf with 1 wt% VC (orange dots). **b** Time-dependent change of SEI resistance for LiTf cells. A different linear timescale was used for the time shortly after assembly to show *R*_SEI_ during the first 1000 min in a more visible way. **c** Nyquist plot for a 1 M LiTFSI in triglyme cell after assembly. **d** Evolution of the SEI resistance and *RC* over time for the 1 M LiTFSI cell. In (**b**, **d**), stars denote when the *E*_A_ of the SEI semicircle was measured, while crosses denote the change of *RC* of the SEI over time.

To determine the effects of different electrolyte chemistries on the SEI formation and its porosity, different symmetric cells were prepared. We chose triethylene glycol dimethyl ether (triglyme) as a solvent and mixed it with lithium triflate (LiTf) and lithium bis(trifluoromethane)sulfonimide (LiTFSI) salts. In addition, to test the potential beneficial effect of additives, cells with 1 M LiTf and 1 wt% VC were also prepared. Figure 2a shows a zoom in of the Nyquist plot related to the transport through SEI (first semicircle) at high frequencies for symmetric cells with 1 M LiTf in triglyme as well as 1 M LiTf with 1 wt% VC directly after assembly.

A single semicircle with a distinct asymmetry is again visible with *f*_max_ = 1 MHz (Fig. 1a) for cells with 1 M LiTf and 1 M LiTf with 1% VC, a frequency value that is higher, compared to the one observed for LP30 containing cells, indicating a faster ion transport process. The total resistance of the semicircle is roughly two orders of magnitude lower than the ones observed in cells with LP30, also indicating an improved SEI chemistry/morphology interplay. Such lower resistance could be explained by SEI with a more porous structure in which presence of liquid electrolyte would lead to higher overall conductivity of the composite, or a complex ratio between porosity, thickness, and pore connectivity. The addition of 1 wt% VC yielded an even more depleted semicircle with similar total SEI resistance. On one hand, this is an indication that the addition of VC does not affect ion transport through the SEI gravely. On the other hand, it is an indication of the fine changes of the capacitance and thus chemistry of the solid part of the SEI, which is in line with the previous studies^32,35,37^.

For cells with 1 M LiTf (Fig. 2a), the shape of the high-frequency semicircle again resembles the semi-infinite pore model for electronically conducting porous electrode, for which de Levie model has been applied^44^. The activation energy was calculated for cells after roughly 6 months of ageing by using both the combination of extended de Levie-Randels model, as well as by manually fitting a model containing parallel resistor and constant phase element (*R*-*CPE* element) to the high-frequency SEI semicircle, as the first option does not account for a pore size/shape distribution, and therefore cannot accurately model the measured impedance (details in SI). For model containing de Levie element, values of *E*_A,SEI_ = 0.28 eV were obtained (SI, supplementary note 4 and Figure S5b), while for a simple *R*-*CPE* element, *E*_A,SEI_ = 0.14 eV (Figure S5a, SI). Both values are reasonable for a porous SEI with important contribution of liquid ion transport pathways, or liquid/solid composite SEI. In addition, the lower *E*_A,SEI_ = 0.13 eV of cells with VC measured after two months of ageing is indicative of a more porous SEI with higher amount of liquid pathways. For 1 M LiTFSI, *E*_A,SEI_ = 0.27 eV of the interfacial process was determined after six months of cell ageing. The shape of the interfacial semicircle was similar to the one observed in LiTf cells with VC, but with higher resistances. When VC was added to the electrolyte, the evolution of *R*_SEI_ was measured over the first 14 h after the cell assembly. A limited growth could be observed (from 107 Ω to 243 Ω). After that, a decrease is observed, which can be explained by mechanical instabilities of the SEI, followed by exposure of fresh silicon to the electrolyte and the buildup of a new SEI layer.

The resistance over time varied in all cells, while the addition of VC to LiTf yielded a more stable resistance evolution over time. LiTFSI cells showed a roughly ten times higher interfacial resistance than LiTf cells. It should be noted that *R*_SEI_ of LiTFSI cells directly after assembly was only around double of *R*_SEI_ of LiTf cells. As all cells contained the same type and amount of solvent, this change in interfacial resistance of LiTFSI cannot be explained by the cell drying out, and was attributed to an ongoing formation of the SEI, its chemical composition and complex distribution of phases.

To determine the effects of electrolyte concentration, cells with 0.2 M LiTf and LiTFSI were assembled and compared with the previous data collected at higher salt concentration. The as-received commercial triglyme contained ca. 600 ppm H_2_O as measured via coulometric Karl-Fischer titration. To determine the effects of water content in the electrolyte on the SEI formation, as recieved triglyme was dried over molecular sieves and cells with dry electrolyte with and without VC additive were assembled. As the commercial LP30 contained < 1 ppm H_2_O, no attempts were made at drying the electrolyte further.

**Fig. 3:**
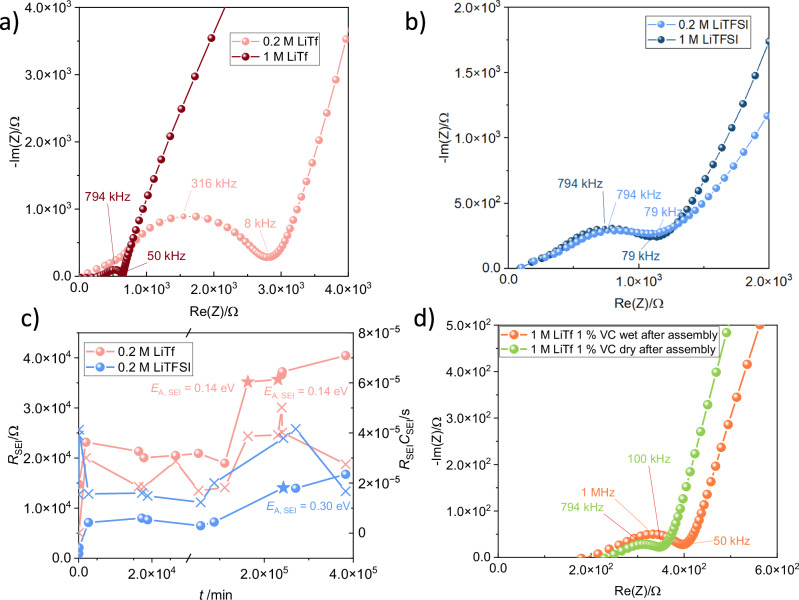
Effects of concentration and water content on the SEI resistance from cells containing symmetric Si electrodes in contact with different triglyme-based electrolytes. **a** Nyquist plots for cells directly after assembly with 1 M LiTf in triglyme (brown dots) and 0.2 M LiTf (pink dots). **b** Nyquist plots for cells directly after assembly with 1 M LiTFSI in triglyme (dark blue dots) and 0.2 M LiTFSI (light blue dots). **c** Time-dependent change of SEI resistance, *R*_SEI_, and time constant, *RC* for cells with 0.2 M LiTf and LiTFSI. **d** Nyquist plots for cells containing 1 M LiTf with 1 wt% VC and 12 ppm H_2_O (dry) and 600 ppm H_2_O (wet) directly after assembly (green and orange dots).

For LiTFSI, no significant difference in cell behavior could be observed for 0.2 M and 1 M salt concentrations (Fig. 3b), while 0.2 M LiTf cells showed around ten times higher interfacial resistance than cells with 1 M LiTf (Fig. 3a). Thus, we can conclude that the relevance of salt-concentration behavior is highly chemistry dependent. However, for both chemistries, a similar depleted SEI-related semicircle could be observed, with an *f*_max_ value between 300 and 800 kHz. The change of SEI resistance over time in cells with 0.2 M LiTf and LiTFSI showed a similar trend as the 1 M cells, with an initial rise of the SEI resistance, followed by a relatively stable period afterwards (Fig. 3c). The *E*_A,SEI_ of the ion transport through the SEI was determined to be 0.14 eV for 0.2 M LiTf and 0.30 eV in the case of 0.2 M LiTFSI. Figure 3d shows a zoomed-in Nyquist plot of the SEI semicircle of wet ( ~ 600 ppm H_2_O) and dry (12 ppm H_2_O) 1 M LiTf cells with VC directly after assembly and after one month of storage. No major differences in the shape and SEI resistance could be observed. In cells containing LiPF_6_ and carbonate-based electrolytes, the reaction of LiPF_6_ and water forms reactive acid and gas^42^, causing additional continuous accelerated reactions with readily available lithium ions, electrons and SEI compounds^48^ through

$${{{\rm{LiP}}}}{{{{\rm{F}}}}}_{6}+{{{{\rm{H}}}}}_{2}{{{\rm{O}}}}\longrightarrow {{{\rm{LiF}}}}+2{{{\rm{HF}}}}+{{{\rm{P}}}}{{{{\rm{F}}}}}_{3}{{{\rm{O}}}}$$

$${{{\rm{P}}}}{{{{\rm{F}}}}}_{3}{{{\rm{O}}}}+{{{\rm{n}}}}{{{{\rm{e}}}}}^{-}+{{{\rm{nL}}}}{{{{\rm{i}}}}}^{+}\to {{{\rm{LiF}}}}\downarrow +{{{\rm{L}}}}{{{{\rm{i}}}}}_{{{{\rm{x}}}}}{{{\rm{PO}}}}{{{{\rm{F}}}}}_{{{{\rm{y}}}}}\downarrow$$

$${{{\rm{HF}}}}\,+\, {\left({{{\rm{C}}}}{{{{\rm{H}}}}}_{2}{{{\rm{OC}}}}{{{{\rm{O}}}}}_{2}{{{\rm{Li}}}}\right)}_{2}+{{{\rm{L}}}}{{{{\rm{i}}}}}_{2}{{{\rm{C}}}}{{{{\rm{O}}}}}_{3}\to {{{\rm{LiF}}}}\\ \,+\, {\left({{{\rm{C}}}}{{{{\rm{H}}}}}_{2}{{{\rm{COC}}}}{{{{\rm{O}}}}}_{2}{{{\rm{H}}}}\right)}_{2}+{{{{\rm{H}}}}}_{2}{{{\rm{O}}}}+{{{\rm{C}}}}{{{{\rm{O}}}}}_{2}\uparrow .$$

Ha et al. reported the formation of pitted SEI structures on Si wafer during OCV rest of carbonate electrolyte containing cells with 50 ppm of water in the electrolyte^40^. Due to the hydrophilicity of Si compared to graphite and the use of aqueous binders, dried electrodes might still have a significant amount of adsorbed water. LiTFSI and LiTf salts are more tolerant towards water, as they are highly soluble and thus used in water-in-salt electrolytes^49–51^. This is beneficial for electrode manufacturing, as it has been demonstrated that standard drying methods cannot remove all adsorbed water on Si electrodes^48^.

**Fig. 4:**
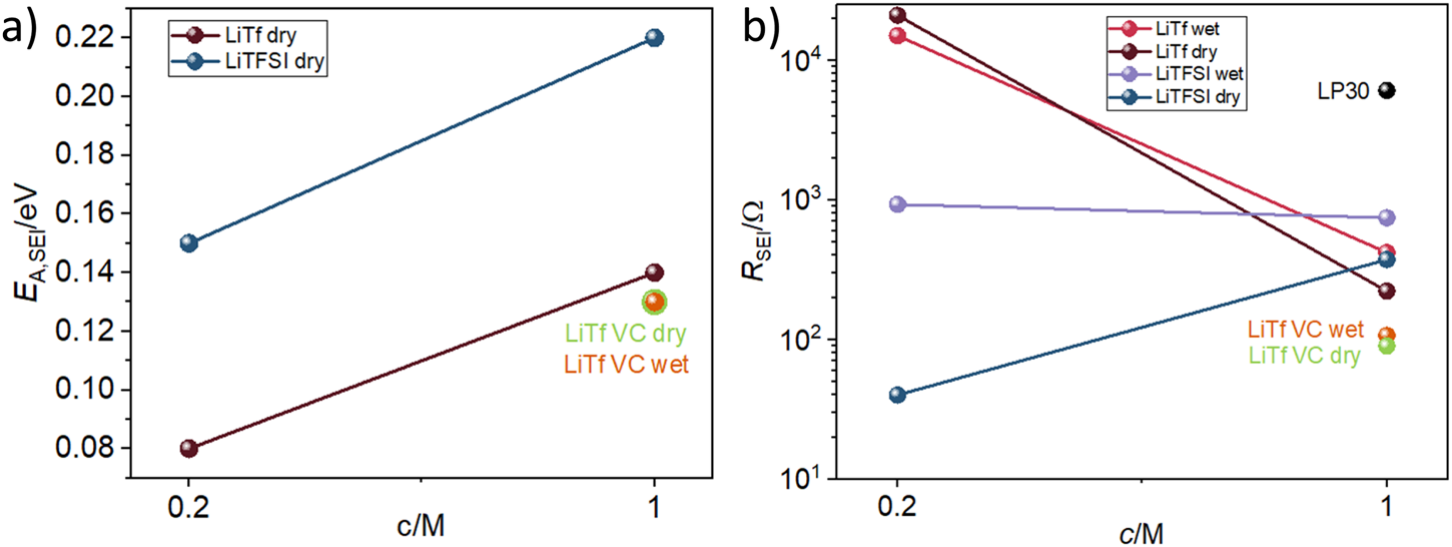
Overview of concentration dependence of measured activation energies, *E*_A,SEI_, and resistance of the SEI, *R*_SEI._ **a** *E*_A,SEI_, and (**b**) *R*_SEI_, in cells with triglyme solvent, different lithium salts, vinylene carbonate as an additive, and varied water content after two months of storage.

Finally, we compare the measured *E*_A,SEI_ for symmetric cells containing triglyme solvent after 2 months (Fig. 4a) in detail. The measured activation energies are all in the range of what is expected for liquid/solid composite or porous SEIs infiltrated with liquid electrolytes, while their values vary between 0.08 eV and 0.22 eV. This means that they either are fully dominated by liquid ion transport at the lower value bound, *E*_A,SEI_ ≈ *E*_A,electrolyte_ or are already slightly densified so that both ion transport from the liquid phase and from the solid phase are relevant, *E*_A,liquid_ < *E*_A,SEI_ < *E*_A,solid_^32^. A trend of *E*_A,SEI_ increase with concentration could be observed indicating a less porous SEI for LiTf and LiTFSI. However, it should be noted that the effect was only minor and all SEIs still displayed a high relevance of liquid ion transport pathways, with values never exceeding 0.2 eV.

Figure 4b shows the initial *R*_SEI_ value (directly after cell assembly) against salt concentration for all cells. A logarithmic scale was used due to the large differences in the SEI resistance between different chemistries. For LiTf, high resistances are observed in both wet and dry cells, while the addition of VC lowered the *R*_SEI_ value. As *E*_A,SEI_ of LiTf cells increased with concentration, which could be explained by more salt in the electrolyte forming a more inorganic SEI and/or one with less liquid interactions with a higher *E*_A,SEI_^31^ a similar rise in resistance could be expected with concentration. It is therefore unclear why *R*_SEI_ decreased drastically with this concentration change. In the case of LiTFSI, dry cells showed significantly reduced SEI resistances. This indicates that small traces of water in the solvent may have an effect on the SEI morphology in cells with triglyme, causing SEI densification which would increase both *E*_A,SEI_ and *R*_SEI_ values.

## Conclusions

In this paper, we showed the time-, concentration and electrolyte chemistry dependent behavior of a composite liquid/solid SEI formed on silicon electrodes in contact with carbonate and glyme based electrolytes. This liquid/solid composite nature of the SEI was observed as a sloped EIS semicircle with *E*_A,SEI_ values varying from 0.08 eV to 0.32 eV, indicating that ion transport through the liquid phase is either dominating or relevant to a significant degree. Even under near-to-OCV conditions, silicon cannot be treated as an inert electrode in contact with carbonate and glyme electrolytes.

We show a strong dependence of the SEI resistance in triglyme cells with LiTf salt, with a roughly tenfold increase at lower concentrations (0.2 M), while LiTFSI showed less variation. The shape of the SEI semicircles stayed similar for both chemistries, indicating a comparable SEI morphology/chemistry combination. A slight overall correlation of *E*_A,SEI_ and salt concentration was visible.

The presence of ~600 ppm H_2_O in commercial triglyme increases the SEI resistance in both with LiTf and LiTFSI salts and causes a higher *E*_A,SEI_, indicating a higher volume of solid in the solid/liquid composite, compared to the dry electrolyte situation. Since denser SEI could be beneficial for its growth mechanism, and the resistances are not gravely increased, water addition, when controlled, could even be beneficial for overall performance. In addition, we confirm the positive effect of VC addition even under close-to open circuit conditions as in 1 M LiTf in triglyme it caused more stable SEI resistance values over time.

Determination of the SEI morphology and thickness might be possible using neutron reflectometry and will give further insights into the structure of the porous SEI. A follow-up study using deuterated solvents will be performed to study the effects of salt, concentration and time on the SEI. In the future, here presented methodology can be used to study the influence of galvanostatic/potentiostatic treatment on the SEI formation. This is of relevance for the potential forming processes in cells containing Si or Si-containing anodes. In addition, the influence of lower overpotentials in EIS measurement could be studied to determine their effects on SEI. At lower measurement frequencies, where diffusion processes can be observed, the overpotential behaves more like a DC polarization, possibly affecting the cell. In this work, planar silicon wafers serve as a simplified model system and are not practical electrodes for commercial batteries. Thus, the use of another model system with a known and well-defined porosity, such as Si wafers with etched pores of defined geometry or porous Si electrodes with known morphology, could serve as an important crosslink between planar systems and commercial Si anodes.

## Methods

### Materials preparation

The preparation of electrolytes and cells was performed in a glovebox (<1 ppm H_2_O, < 1 ppm O_2,_ Vacuum Technology Inc.) with an Argon atmosphere (Ar 5.0, Nippon Gases). Liquid electrolytes were prepared by dissolving Li-salts (LiTf: 98%, TCI; LiTFSI: Sigma-Aldrich, n/a purity) and vinylene carbonate (97%, Sigma-Aldrich) in triglyme (99%, Thermo Scientific). LP30 (1 M LiPF_6_ in ethylene carbonate/dimethyl carbonate 50/50 v/v, battery grade, Sigma-Aldrich) was used as received. The H_2_O content of the as received triglyme was measured to be 599 ppm using coulometric Karl Fischer titration (Nittoseiko Analytech CA-310), the water content of LP30 was below 1 ppm. For electrolytes with dry solvent, the as received triglyme was stored over 3 Å molecular sieves for 72 h. The measured H_2_O content upon treatment was 12 ppm.

### Electrode and cell preparation

9.9 mm diameter silicon electrodes were cut out of a silicon wafer (single side polished, <111 > , N-type, no dopant, 2-inch x 0.5 mm, Sigma-Aldrich) using a laser cutter in air. When assembling symmetric cells, the polished side of the Si wafer faced the separator. For the separator, 11 mm diameter discs were punched out of GF/B glass microfiber filters (55 × 0.5 mm, Whatman). For assembly of the symmetrical coin cells, a 1 mm steel spacer was used for a total stack height of 2.5 mm. No spring was used due to the fragility of the Si electrodes. After assembly, the sealed coin cells were taken out of the glovebox and stored in air at RT.

### Electrochemical measurements

For electrochemical measurements, the coin cell was placed in a modified SMD (surface mount device) impedance fixture. Electrochemical impedance analysis (EIS) was carried out in the potentiostatic mode in the frequency range from 10^7^ to 10^−^^3^ Hz using Novocontrol Alpha-N and Novocontrol Beta-N dielectric analyzers. The impedance fixtures were connected to the dielectric analyzers using a multiplexer, consisting of a HP 3499 A Switch/Control System and HP 44472 A VHF switch modules. The total cable length for each impedance fixture was kept constant at 2 m, while the inbuild cable correction compensation of the dielectric analyzers was used to account for the impedance of the cables/setup. For measurements above 10 MHz, the impedance fixtures were directly connected to the Novocontrol Beta-N device. All measurements were performed with 10 mV amplitude. EIS data analysis was performed using the ZView software from Scribner Associates, version 4.0 h. Kramers-Kronig relations were used to check for nonlinearity and instability, only data ranges that complied were used for fitting.

When needed, the temperature of the cells was externally controlled by a Lauda Pro 2090 thermostat. The impedance fixture was placed inside a semi-submerged aluminum box in the ethylene glycol/water bath. The temperature of the water bath was controlled by the thermostat. Measurements were taken 30 min after the bath temperature reached the set value to ensure thermal equilibration.

## Supplementary information

Supplemental Material

## Data Availability

The authors declare that the data supporting the findings of this study are available within the paper and its Supplementary Information files. Raw data is available on Zenodo: 10.5281/zenodo.14212964.
