# Supplementary material for: Long term porosity of solid electrolyte interphase on model silicon anodes with liquid battery electrolytes
Source: Commun Chem. 2024 Dec 19;7:297. doi: 10.1038/s42004-024-01381-2 (PMC11659429; doi:10.1038/s42004-024-01381-2)
Supplement: Supplementary file 1 — Supplemental Material [file 42004_2024_1381_MOESM1_ESM.pdf]

Supplementary Information for

Long term porosity of solid electrolyte interphase on model silicon anodes with liquid battery electrolytes

Jonas Grill, Jelena Popovic-Neuber*

Department of Energy and Petroleum Engineering, University of Stavanger, Norway

Corresponding author: jelena.popovic-neuber@uis.no

Table of contents

Supplementary Note 1: Calculation of activation energies E_A from temperature-dependent EIS

Supplementary Note 2: Considered equivalent circuits

Supplementary Note 3: Impedance response of porous electrodes[1]

Supplementary Note 4: Fits of the EIS data

Supplementary Note 1: Calculation of activation energies E_A from temperature-dependent EIS

Activation energies of electrochemical processes observed as semicircles in Nyquist-plots can be calculated by plotting *T/R* [KΩ^{-1}] against 1/*T* [K^{-1}], with the resistance *R* of the process

$$\ln(\sigma T) = \ln(\sigma_0) - \frac{E_A}{k_B} \cdot \frac{1}{T} \text{ (Eq. 1)}$$

with the ionic conductivity σ, the pre-exponential factor σ_0, the temperature *T*, Boltzmann constant k_B and activation energy E_A. E_A can then be obtained as the slope of a linear fit in units of $1/k_B$.

Supplementary Note 2: Considered equivalent circuits

The equivalent circuits used to model the data consist of the electrolyte resistance R_{el}, followed by either a parallel RC-circuit to model the observed SEI semicircle (consisting of the SEI resistance R_{SEI} and its capacitance C_{SEI}, as shown in Fig. S1a) or a de Levie element, marked as "L" (Fig. S1b). De Levie element is described in detail in part 3 of this SI. The RC-element for the SEI is described in detail in our earlier work on porous SEIs on Li and Na electrodes[2]. As the observed $E_{A,SEI}$ was comparable to $E_{A,liquid}$, R_{SEI} describes the resistance of liquid electrolyte in pore channels, C_{SEI} described the capacitance of the solid part of the SEI and R_{el} describes the

resistance of the bulk electrolyte (see figure 1d in reference [2] for a more detailed explanation). Charge-transfer and diffusion processes are described by a Randles circuit with the charge-transfer resistance R_{CT}, double-layer capacitance C_{DL} and Warburg impedance W[3]. As Li^+ cannot enter Si under near OCV conditions, blocking electrodes were assumed and a Warburg-open element was used during fitting. Prior to fitting, Kramers-Kronig relations were used to check the linearity and accordingly datapoints at the highest frequencies from 10 MHz to ~3 MHz were emitted. Similarly, data below 10 mHz were also emitted from the fits. The electrolyte resistance R_{el} was read from the x-axis intercept and used a fixed parameter in the fits.

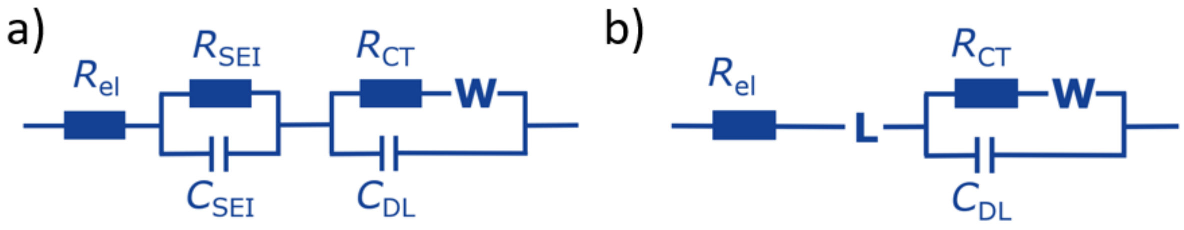

Figure S1. Equivalent circuits used for fitting of the measured EIS data: a) SEI described as an RC-element, b) SEI described by a de Levie model for a porous electrode.

Supplementary Note 3: Impedance response of porous electrodes[1]

The most fundamental equivalent circuit model of the interface of electrodes in EIS consists of an RC circuit, with the resistance of the SEI R_{SEI} and its capacitance C_{SEI} in parallel. This model was developed for ideally polarizable liquid electrodes and describes a perfect semicircle in the Nyquist plot. The impedance Z can be described as

$$Z = \frac{1}{\frac{1}{R_{SEI}} + j\omega C_{SEI}} \text{ (Eq. 2)}$$

where j is the imaginary unit and $\omega = 2\pi f$ with the frequency f.

As batteries use solid electrodes where the double-layer capacitance is not purely resistive (i.e. a "leaking" capacitor), the constant phase exponent ϕ is introduced as the deviation from the straight 90° capacity line in the Bode plot

$$Z = \frac{1}{\frac{1}{R_{SEI}} + (j\omega)^{\phi} T} \text{ (Eq. 3)}$$

where the parameter T is related to the capacitance [F $s^{\phi-1}$ cm^{-2}]. This parallel combination of a resistance and a constant phase element (CPE) yields a depleted or “sunken” semicircle with their center being located below the real axis. The further ϕ decreases from an ideal capacitor (ϕ = 1), the more depleted the semicircle. This model is usually sufficient to describe the electrochemical impedance of planar electrodes, with the electrolyte resistance R_{el} in series to the R-CPE circuit of the electrode.

For porous electronically conducting electrodes, an asymmetry of the semicircle can be seen in the Nyquist plot, which depends on the morphology of the pores, such as their pore depth to diameter ratio l/r. De Levie developed an equation for the impedance of a cylindrical pore, with the assumptions that the pore is filled by electrolyte, only the side walls are conducting and there exists an AC potential gradient in the axial direction. The impedance of a pore can then be described as

$Z_{pore} = \frac{R_{\Omega,p}}{\Lambda^{1/2}} \coth\left(\Lambda^{1/2}\right)$ (Eq. 4) and

$\Lambda = \frac{2\rho_s l^2 j\omega C_{dl}}{r}$ (Eq. 5),

with the total resistance of the pore $R_{\Omega,p}$, the dimensions admittance of the pore Λ, the resistivity of the solution in the pore ρ_s, the pore length l and radius r. The abovementioned model describes the impedance of a porous electrode if the AC signal penetration length is comparable to the length of the pore. At high frequencies, a coupling of solution resistance and double-layer capacitance is observed as a 45° slope, followed by a semicircle at lower frequencies. For shallower pores, the AC signal can always penetrate to the bottom of the pore at all frequencies, resulting in a perfect semicircle. For deep and narrow semi-infinite length pores with a large l^2/r ratio the AC signal penetration length λ might be smaller than the pore length. The equation for the pore impedance can be simplified with $\Lambda \to \infty$, $\coth(\Lambda^{1/2}) \to 1$ yielding

$Z_{pore} = \frac{R_{\Omega,p}}{\Lambda^{1/2}}$ (Eq. 6).

In this case, the Nyquist plot contains a straight 45° line at high frequencies, followed by a skewed semicircle. If the capacitive element is substituted for a constant phase element, it can be written as:

$$Z_{pore} = \frac{1}{\sqrt{\frac{1}{A} + (j\omega)^{\phi}T}}$$ (Eq. 7), where

$$A = \frac{R_{CT}\rho}{2\pi^2 r^3}$$ (Eq. 8).

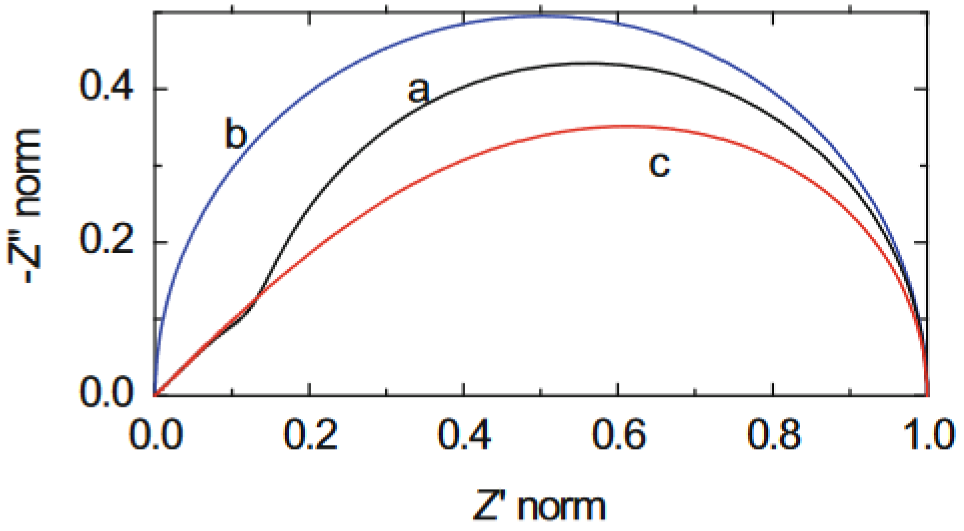

Figure S2. Normalized Nyquist plots (Z/Z_{max}) for a porous electrode in presence of redox species. a) general case, AC penetration length $\lambda \sim l$, b) shallow pore, $\lambda >> l$, c) semi-infinite pore, $\lambda << l$. Reprinted with permission from Ref. [1].

Lasia simulated the effect of the overpotential on the Nyquist plot[4], Figure S2 shows normalized Nyquist plots of a porous electrode for the following three scenarios: with small overpotentials the potential drop in the pore is minimal, leading to almost ideal semicircles (see Fig S2, case b). At higher overpotentials a 45° slope at high frequencies was observed (Fig S2, case a), followed by a sloped semicircle for even higher overpotentials (Fig S2, case c). This could serve as an additional test to confirm the existence of a porous electrode. As the aim of this paper was the observation of OCV SEI formation, this approach was only tested with overpotentials between 2 and 20 mV_{rms} (root mean square voltage). At lower overpotentials, noise overwhelmed the measured data, while higher potentials push the cells further away from OCV conditions and may stray out of the pseudo-linear region of the cells. No significant differences could be observed between 2 and 20 mV_{rms} overpotential. As λ depends on the overpotential and the pore geometry is unknown, only the product of the experimentally accessible parameters, $R_{ct}C_{dl}$ can be extracted. For the equivalent circuit models, the equations related to the model which fit best visually (general case, shallow pore, semi-infinite pore) was used. If a perfect semicircle was observed, a parallel R-CPE model was used. This allows for qualitative assessment of the pore geometry.

Supplementary Note 4: Fits of the EIS data

Table S1 lists the fit parameters of the fits shown in Figure S3. The fitting seems to be suitable in the medium frequency regime but deviates from observed data for low frequencies (i.e. Warburg response). While the error values of R_{SEI} were relatively low, fitting the Randels circuit proved challenging as fits of the Warburg impedance produced error ranges of over 100% for the terms related to resistance and capacitance (Wo-R and Wo-T, table S1) in some cases (see table S1). In some cases, values for the CPE in the Randels circuit had to be fitted with all other parameters fixed (and vice versa) as the fits would otherwise converge on non-sensical values.

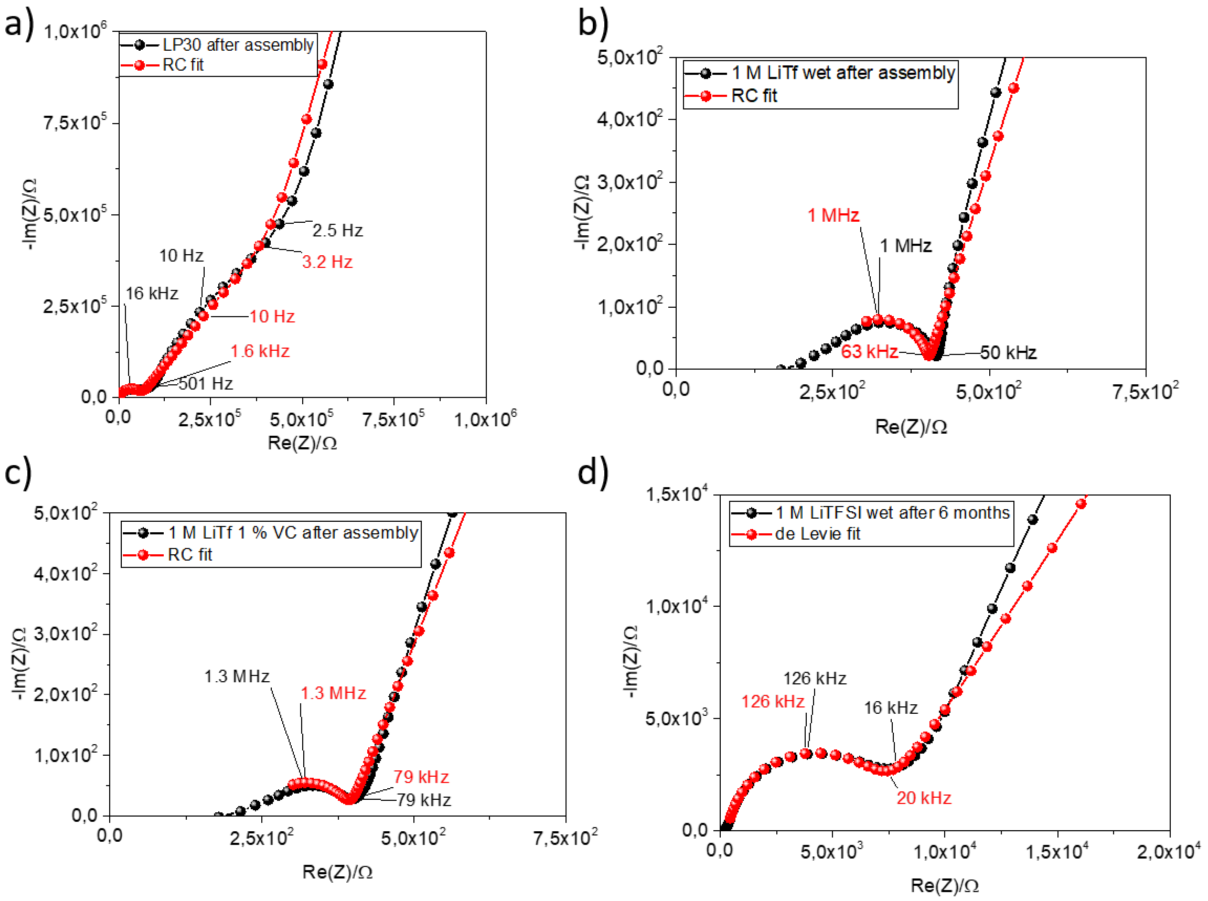

Figure S3. Zoomed-in Nyquist plots of the SEI semicircle for different electrolytes, with fits of the equivalent circuit models described in chapter 1: a) Nyquist plot of a LP30 cell after assembly and the corresponding fit with an RC model (see fig S1 a for the equivalent circuit model), b) Nyquist plot of a 1 M LiTf cell after assembly and the corresponding fit with an RC model, c) Nyquist plot of a 1 M LiTf cell with 1 % VC after assembly and the corresponding fit with an RC model, d) Nyquist plot of a 1 M LiTFSI cell six months after assembly and the corresponding fit of the SEI semicircle with the de Levie model (see fig S1 b for the equivalent circuit).

Table S1. Parameters of the fits shown in Figure S3. SEI resistance R_{SEI}, SEI capacitance C_{SEI}-T of the constant phase element, constant phase exponent ϕ, charge-transfer resistance R_{CT}, Warburg-impedance Wo1-R, capacitance Wo1-T and constant phase exponent ϕ, double-layer capacitance CPE-C_{DL} and constant phase exponent CPE-CDL-P. For fits with the de Levie element, LS1-A describes the admittance term (see eq. 5), capacitance term LS1-B, resistance LS1-R and constant phase exponent LS1-Phi. Errors of all fitted parameters are given as (+/- %).

	Chi-Sqr	Rel(X)	RSEI(+)	RSEI(Error%)	CSEI-T(+)	CSEI-T(Error%)	CSEI-P(+)	CSEI-P(Error%)	RCT(+)	RCT(Error%)
**LP30**	2,5E-03	137	57733	2,7	1,62E-09	10,5	0,81	1,0	33015	227,3
**1 M LiTf**	1,8E-03	248	152,2	2,8	7,84E-10	85,0	1,02	5,4	30810	29,4
**1 M LiTf 1 % VC**	1,2E-03	248	145,8	2,8	1,92E-08	80,3	0,81	6,4	42359	48,9

	Wo1-R(+)	Wo1-R(Error%)	Wo1-T(+)	Wo1-T(Error%)	Wo1-P(X)	CPE-CDL-T(+)	CPE-CDL-T(Error%)	CPE-CDL-P(+)	CPE-CDL-P(Error%)
**LP30**	2,64E+06	8,9	0,22759	9,9	0,5	1,52E-07	5,4	0,65	2,3
**1 M LiTf**	5,70E+06	65,9	479,3	131,4	0,5	1,99E-06	8,4	0,82	1,3
**1 M LiTf 1 % VC**	6,00E+06	72,7	242,4	145,9	0,5	2,92E-06	7,7	0,77	1,2

	Chi-Sqr	Rel(X)	Ls1-A(±)	Ls1-A(Error%)	Ls1-B(±)	Ls1-B(Error%)	Ls1-Phi(±)	Ls1-Phi(Error%)	Ls1-R(±)	Ls1-R(Error%)
**1 M LiTFSI**	5,5E-03	80	8,289	20,3	2,85E-07	24,9	0,95482	2,5239	761,4	18,8

	RCT(X)	Wo1-R(±)	Wo1-R(Error%)	Wo1-T(±)	Wo1-T(Error%)	Wo1-P(±)	Wo1-P(Error%)	CPE-CDL-T(X)	CPE-CDL-P(X)
**1 M LiTFSI**	6,83E+06	1,13E+07	25,9	22,15	36,7	0,42636	4,7	2,98E-07	0,64

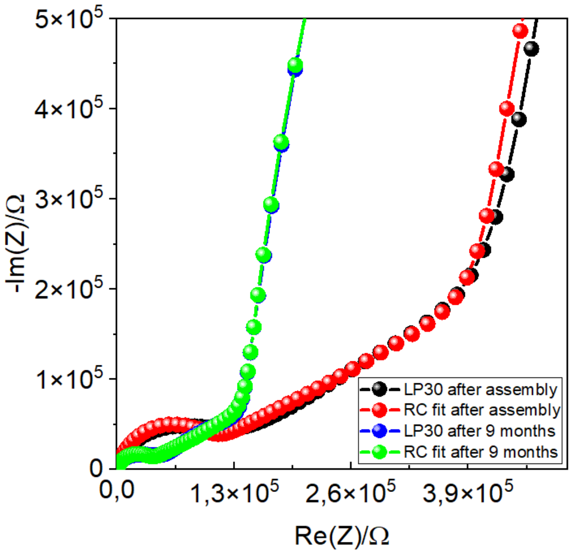

Figure S4. Zoomed in Nyquist plots of the fits for LP30 cells after assembly and after 9 months, using the equivalent circuit model from Figure S1a.

For the calculation of E_A, the model that produced visually better fits was used (either a R-CPE model or a model containinf semi-infinite pore length de Levie element). With de Levie elements, the parameter A containing the resistance was used in place of R_{SEI}. Figure S5 shows a comparison between an impedance spectrum of a 1 M LiTf cell at 65 °C where the SEI semicircle was either fitted with a R-CPE or a de Levie element.

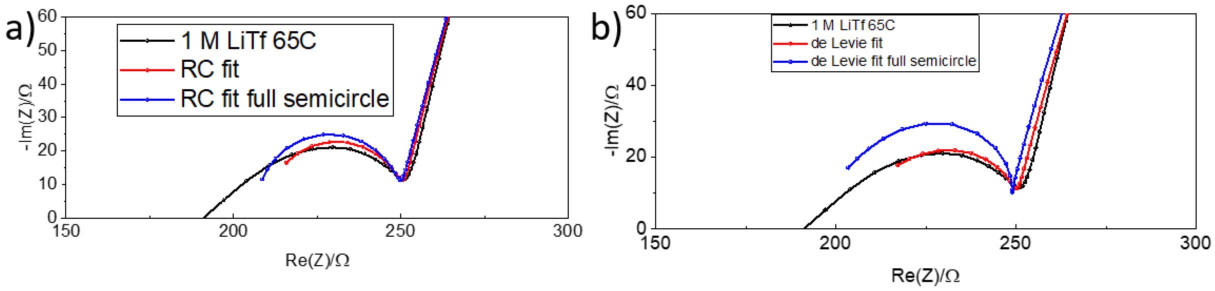

Figure S5. Zoomed in Nyquist-plots of the SEI semicircle in a 1 M LiTf cell at 65 °C. a) SEI semicircle fitted with a R-CPE element (red dots for reasonable fit, blue dots for fitting the entire semicircle), b) SEI semicircle fitted with a de Levie element (red dots for reasonable fit, blue dots for fitting the entire semicircle).

A de Levie model was chosen as it described the asymmetry of the semicircle better, in figure S5a (red dots) the left side of the fitted semicircle did not match the curvature of the measured spectrum. Inclusion of the heavily sloped high frequency part of the SEI semicircle yielded fits where both the frequencies and capacitances deviated strongly from the measured data. The blue dots in Figure S5 denote fits where a fit of the entire semicircle was attempted.

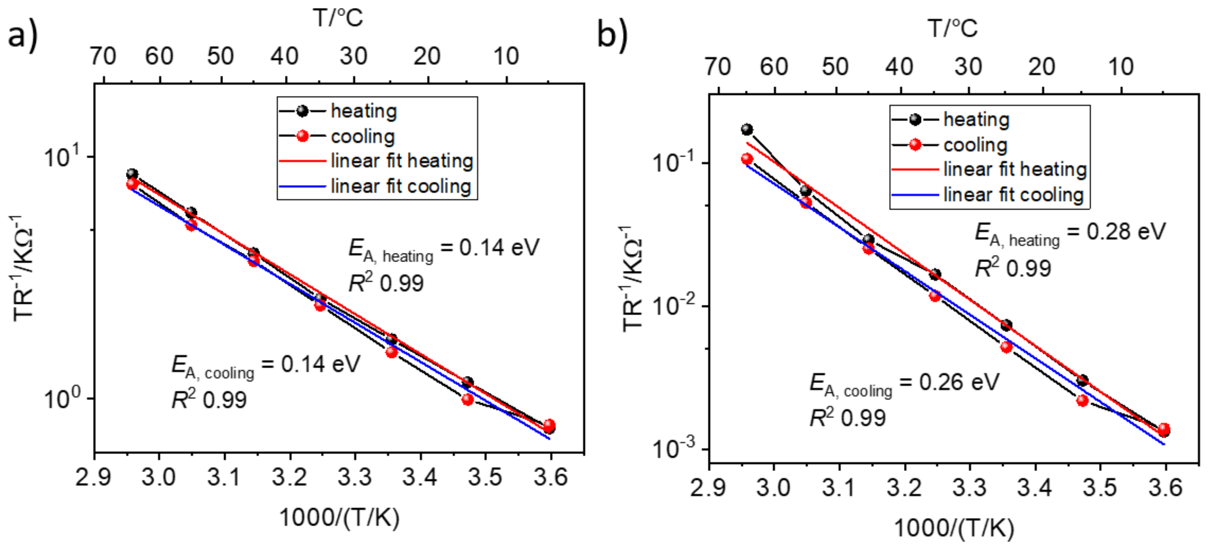

Figure S6. Arrhenius-plots of R_{SEI} of 1 M LiTf cells, fitted with two different equivalent circuit models. a) data fitted with a R-CPE element, b) R_{SEI} from fits with a de Levie model.

Figure S6 shows Arrhenius plots for the determination of $E_{A,SEI}$ of 1 M LiTf cells, with R_{SEI} extracted from different equivalent circuit models. For both models, $E_{A,SEI}$ extracted from heating and colling the cells ($E_{A,heating}$ and $E_{A,cooling}$) aligned well with each other and gave good R^2 values. However, the E_A calculated from fitting a R-CPE model was half of that calculated from fitting a de Levie element containing model. As previously discussed, this was due to the asymmetry of the SEI semicircle.

For cells with LP30, the charge-transfer resistance was extracted from the fits. Figure S7 shows R_{CT} over time, calculated from fitting impedance data with the model shown in Figure S1a. Apart from a single measurement two hours after cell assembly, it remained roughly constant over the course of nine months.

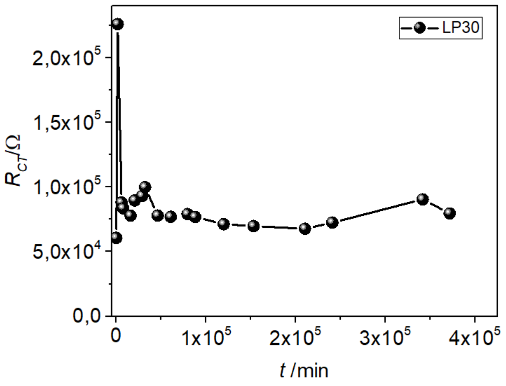

Figure S7. Charge-transfer resistance of LP30 cells over time, calculated by fitting with the model shown in Figure S1a.

References

1. Lasia A. Electrochemical impedance spectroscopy and its applications.). Springer (2002).

2. Lim K., Popovic J., Maier J. Ion transport and growth behavior of solid electrolyte interphases on Li and Na with liquid electrolytes based on impedance analysis. *Journal of Materials Chemistry A* **11**, 5725-5733 (2023).

3. Orazem M. E., Ulgut B. On the Proper Use of a Warburg Impedance. *Journal of The Electrochemical Society* **171**, 040526 (2024).

4. Lasia A. Impedance of porous electrodes. *Journal of Electroanalytical Chemistry* **397**, 27-33 (1995).
